# Features and Outcomes In Utero and after Birth of Fetuses with Myocardial Disease

**DOI:** 10.1155/2010/628451

**Published:** 2010-10-03

**Authors:** Vlasta Fesslova, Maurizio Mongiovì, Salvatore Pipitone, Jelena Brankovic, Laura Villa

**Affiliations:** ^1^Center of Fetal Cardiology, Policlinico San Donato IRCCS, Via Morandi 30, San Donato Milanese, 20097 Milan, Italy; ^2^Department of Pediatric Cardiology, “Casa del Sole” Hospital, 90136 Palermo, Italy; ^3^First Obstetric and Gynecologic Clinic, University of Milan, 20122 Milan, Italy

## Abstract

*Objectives*. Ninety-one fetuses with dilated or hypertrophic cardiomyopathy (DCM, HCM) and myocarditis were studied. *Results*. *Group 1 “DCM”* included 19 fetuses: 13 with hydrops (FH) and 5 with associated extracardiac anomalies (ECAs) (15.8%). *Group 2 “Myocarditis”* included twelve fetuses, having 11 with FH. *Group 3 “HCM”* included sixty fetuses: 26 had associated ECAs, 17 had maternal diabetes, and 17 were “idiopathic”; however, in one case, a metabolic disorder was found postnatally, and 4 had familiarity for HCM. *Outcomes*. Ten cases opted for termination of pregnancy. Two cases with DCM and 1 with HCM were lost at follow-up. Out of the cases that continued pregnancy, with known follow-up, mortality was 68.75% in Group 1, 63.6% in Group 2, and 31.3% in Group 3 (the majority with severe ECAs). Surviving cases with DCM and myocarditis improved, 2 with HCM worsened, 6 remained stable, and 26 improved or normalized. *Conclusions*. Our data show more severe prognosis in DCM and myocarditis and forms with severe associated ECAs.

## 1. Introduction

Myocardial disease (MD) is a group of diseases with very variable forms, etiology, entity, and natural history that are mostly diagnosed in adolescence and adulthood [1]. It was reported to occur in about 2–7% in the series of neonates and infants [2, 3]. Fetal echocardiography allows now an early recognition of MD, based upon the pattern of dilated and hypocontractile ventricles or of a various degree and localization of parietal hypertrophy. The frequency of MD in fetal life is difficult to establish—we can consider the figures given in some fetal series: in our experience it was found in 1–1.7% of two series of pregnancies at risk for CHD studied by fetal echocardiography, while its prevalence was 6.7%, 7.5%, and 8.9% of cases with CHD [4–7]. 

Echocardiographic features and outcomes in utero of cases with MD have been described in some studies [8–10], however the data are very variable, being often included also cases with some specific conditions like twin-to-twin transfusion syndrome or cases associated with anemia. In the era of fetal echocardiography, the referral for familial MD is quite frequent and the counseling in these cases is difficult. Therefore we feel it is of interest to have more data regarding this pathology.

## 2. Objectives of the Study

The aim of this paper is to analyse retrospectively our experience regarding the characteristics and outcome of cases with myocardial disease, detected at fetal echocardiography.

## 3. Material and Methods

Between 1990 and 2005, 91 fetuses (1.5%), out of around 6000 cases, referred for fetal echocardiography in 2 centers presented with a pattern of dilated or hypertrophic cardiomyopathy (DCM, HCM), at 17–38 weeks' gestation, median 28. The fetuses were referred often for a finding of abnormal cardiac features or of a fetal hydrops at obstetric scan: some came for a family history of HCM or for maternal diabetes; and, also, the fetuses with extracardiac anomalies were sent for an evaluation of the cardiac state. Cases with twin-twin transfusion syndrome or anemia, as well as those associated with specific congenital heart lesions were excluded from this study. Infections, extracardiac anomalies (ECAs) and maternal disease were always checked.

### 3.1. Methodology

Two-dimensional echocardiography with M-mode and pulsed/continuous wave and Color Doppler were performed using the echocardiographic machines: Acuson Sequoia, Imagegate, Siemens, Erlangen, Germany, and Vivid 7, General Electrics, Healthcare, Italy, with transducers 5 and 3.5 MHz according to the fetal age.

Measurements of the right and left ventricles were done in 2D in 4-chamber view in diastole (at the level of the mitral and tricuspid valve anulus) and in M-mode in a horizontal 4-chambers view, measuring the thicknesses of the right ventricular anterior wall, of the interventricular septum and of the left ventricular posterior wall. Shortening fraction of the left ventricle was calculated on the basis of the M-mode measurements in systole and diastole (normal values 28–42%).

Pulsed and Color Doppler examinations were always performed, and, specifically, pulsed Doppler waveforms were analysed at the level of the atrioventricular and semilunar valves and at the level of the inferior vena cava, ductus venosus, and umbilical artery.

We used as a criterion for the diagnosis of DCM the ventricular enlargement above the 97.5% according to the normal standards for gestational age [11–13] without thickening of the walls and with reduced contractility (shortening fraction <28%).

The term of the “noncompaction” of the left ventricular myocardium was used in cases that showed dilated ventricle with reduced function and numerous prominent trabeculations with deep myocardial recesses [14–16]. HCM was diagnosed on the basis of an increased parietal thickness above 97.5% of normal standards for gestational age [17, 18]. In all cases, congenital heart anomaly was excluded.

Fetal hydrops was defined by the presence of serous effusion in at least two compartments: of a mild degree, when in 2 compartments (ascites+ hydropericardium or hydrothorax), of a moderate degree when in 3 compartments (ascites, hydrothorax, and hydropericardium), and severe, with associated skull edema.

After the diagnosis was made, all cases were screened for possible infections (parvovirus, Coxsackie, cytomegalovirus, and toxoplasmosis), fetal anemia, and diabetes. The fetal rhythm was always evaluated.

### 3.2. Postnatal Assessment

In the cases born alive echocardiography was performed after birth, usually within the first day and repeated subsequently according to the clinical necessity (median follow-up: 6 years; range: 2–14). Methodology of the examination was performed according to the standard criteria, and M-mode measurements were used for functional assessment, as exposed for the fetal examination, comparing the data with normal standard values for the infants' weight and body surface area [19]. All infants underwent a complete check-up of laboratory tests, to exclude underlying conditions as metabolic disorders, infections, and so forth.

### 3.3. Analysis of the Data

The characteristics of the cases and their course in utero and after birth were analysed, according to the type: Group 1-DCM, Group 2-documented myocarditis, and Group 3-HCM.

### 3.4. Statistical Analysis

Gestational age at presentation in cases that died and those who survived was compared by means of Student's test *t* for unpaired data, for single groups.

Influence of hydrops in Groups 1 and 2 and of extra cardiac anomalies in Group 3 on outcome was tested by Student's test *t* for paired data, and the differences in outcomes in categories with and without hydrops in Groups 1 and 2 were compared by chi-square test.

## 4. Results

### 4.1. Features

***Group 1* (see Table 1)**

Pattern of DCM with dilated left ventricle was found in 18 fetuses, associated in 5 (15.8%) with extracardiac anomalies. One fetus had a form with dilated right ventricle, associated with an abnormal tricuspid valve apparatus.

**Figure 1:**
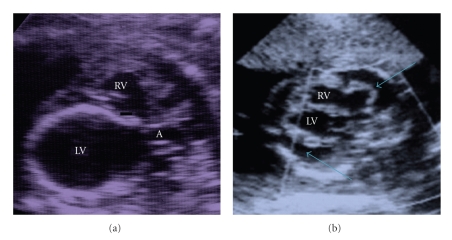
(a) Echocardiography of a fetus with endocardial fibroelastosis—a high echodensity of the left ventricular (LV) walls due to calcifications is evident. RV—right ventricle, A—aorta. (b) Fetus with myocarditis and fetal hydrops: both ventricles and atria are dilated and the arrows indicate the pericardial effusion. RV—right ventricle, LV—left ventricle.

Three fetuses presented a pattern of noncompacted myocardium (2 siblings), and 8 fetuses had a highly echodense endomyocardium, suggesting endocardial fibroelastosis (3 with ECA), with calcifications in one case (Figure 1(a)) that were documented also at postnatal X-ray and at autopsy following the death in the 1st day of life. One fetus had a family history of cardiomyopathy, in a 3-year-old sibling affected with a mild form.

Thirteen cases (68.4%) showed at presentation fetal hydrops: 12 of a moderate-to-severe degree and 1 of a mild degree.

**Cardiac Function at Echocardiography**

All cases had reduced contractility—with fractional shortening ranging between 10 and 20%, median 16. An abnormal pattern of systolic and diastolic flows of the mitral and tricuspid valves (reduced E/A ratio and a small A-wave) and atrioventricular valves regurgitation were seen at Echo-Doppler. All fetuses showed mitral regurgitation of a mild-moderate degree, and the 13 cases with hydrops presented moderate-severe holosystolic tricuspid regurgitation, associated with anomalous flow of the inferior vena cava and ductus venosus (a reverse A-wave flow in systole) and the fluctuation of the umbilical venous flow in cases in preterminal condition.

***Group 2* (see Table 1)**

Twelve fetuses with documented viral infection (5 with Cytomegalovirus and 7 with Coxsackie virus) presented dilation of both ventricles (see Figure 1(b)), with tricuspid and mitral regurgitation and fetal hydrops in 11 cases (91.7%), of a severe degree in 5 cases, of a moderate degree in further 5 cases, and of a mild degree in one case.

**Figure 2:**
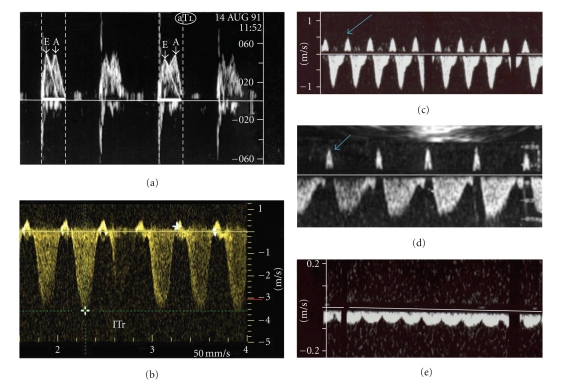
Doppler findings in fetuses with DCM and fetal hydrops: (a) The tracing of the tricuspid valve (T) shows a reduced systolic A-wave, with respect to the diastolic E-wave; (b) holosystolic tricuspid regurgitation (ITr); (c) a reverse systolic flow of the inferior vena cava (small arrow); (d) a reverse systolic flow of ductus venosus (small arrow); (e) the fluctuation of the umbilical vein.

All these case presented abnormal systolic left ventricular or biventricular function and signs of regurgitation of both atrioventricular valves, associated in the cases with fetal hydrops to abnormal venous flows, as described for Group 1 (see Figure 2).

***Group 3* (see Table 2)**

Sixty fetuses presented HCM of the left ventricle, with outflow tract obstruction in two; 10 also presented right ventricular hypertrophy; 26 (43.3%) had extracardiac anomalies (ECAs), 3 cases were affected by syndromes precised after birth (1 Thomas syndrome, 1 Prune-Belly, and 1 Noonan).

Seventeen fetuses (28.3%) had mothers with diabetes (pregestational in 12; gestational in 5); 17 fetuses were considered to be “idiopathic”, however in one of them a metabolic disorder (cytochrome-oxidase deficiency) was found postnatally, while 5 remaining cases presented a family history of HCM.

Systolic function was normal or slightly increased (shortening fraction between 30 and 45%, median 36), some fetuses showed signs of abnormal diastolic flow through the mitral valve (higher E-A velocities) at Echo-Doppler and mild mitral regurgitation. The two cases with left ventricle obstruction had slightly increased Doppler velocity through the aorta (2 and 2.2 m/sec.). Heart failure in utero was infrequent and only mild (in 4/60 cases = 6.7%).

None of the fetuses with hypertrophy had features fitting for the diagnosis of restrictive cardiomyopathy—showing no particular enlargement of atria and no specific Doppler findings of mitral valve.

### 4.2. Outcome and Evolution: (See Tables 1 and 2 and Figure 3 with a Flow Chart)

#### 4.2.1. DCM

Out of 19 cases with DCM, 1 opted for termination of pregnancy (TP), 2 were lost at follow-up, and *total mortality* was 11/16 cases that continued pregnancy and with a known follow-up (68.75%). Eleven out of 13 cases with fetal hydrops died (2 in utero). Two of the 3 cases with noncompacted myocardium died in utero. Surviving cases improved after 4 months–1 year.

#### 4.2.2. Myocarditis

Out of 10 cases with moderate-to-severe hydrops, 1 opted for TP and only 2 survived. One fetus with mild-initial hydrops that presented at 20 w.g. and another one who presented late at 37 w.g. without hydrops survived. 

*Total mortality* was 7/11 cases that continued pregnancy (63.4%) Four cases that survived improved all after 3–12 months.

#### 4.2.3. HCM

**(a) Idiopathic Form**

One out of 5 cases with *familial history* (in mother, grandfather, or siblings) was diagnosed after birth to have a metabolic disorder-cytochrome-oxidase deficiency (as said above) and died at 38 days. The remaining 4 infants are alive at 5–14 years and present mild-moderate forms, one needing recently, at 10 years, propranolol for a moderate left ventricular obstruction.

**Figure 4:**
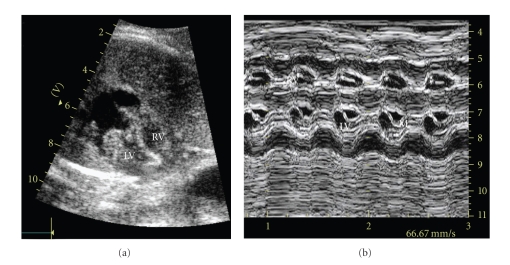
(a) Fetus with hypertrophic cardiomyopathy in 2-D; (b) M-mode recording showing hypertrophy of the left ventricular walls, with a small cavity and mitral valve movement with diastolic apposition and anterior systolic motion. RV—right ventricle, LV—left ventricle, M—mitral valve.

Out of the 12 cases with a *negative family history*, 1 case was lost at follow-up, 1 infant born premature for fetal distress at 28 w.g. died at 15 days and 2 other cases worsened: one with a biventricular obstruction, shown in Figure 4, delivered at 32 w.g., required a heart transplant at 2 months and is alive at 4 years, while the second one, also with a biventricular obstruction since birth, treated with betablockers, developed a severe left ventricular obstruction due to an excessive mitral valve tissue at 3 years and needed a cardiosurgical excision of this tissue together with a plasty of the mitral valve. Two cases remained stable and 6 improved at 3 months–1 year.

**(b) HCM Secondary to ECA**

Eight opted for TP, 6 had IUD (5 renal anomalies-Potter syndrome and 1 Prune Belly). Seven out of the 12 infants that are born alive died after birth and 5 are alive at 5–12 years and improved.

One case with CNS anomaly was operated for craniostenosis and is alive; equally, the infant with Noonan syndrome suffered initially from pleural effusions, stabilizing thereafter.

**(c) HCM Secondary to the Maternal Diabetes**

One fetus of a mother with pregestational diabetes presented at 29 w.g. with mild fetal hydrops and atrial flutter resolved by the maternal-fetal therapy, with digoxin was delivered at term showing only a mild LV dyskinesis after birth, regressed at 1 month.

Two infants with pregestational maternal diabetes died: one with a poor control of diabetes in pregnancy had a severe HCM and died immediately after birth, of cardiac arrest, while the second one, with a borderline thickness of the interventricular septum in the third trimester and immediately after birth, developed progressively a massive parietal hypertrophy, obstruction of the left ventricular outflow and died at 4 months of an untreatable heart failure. All surviving cases with *maternal diabetes* progressively normalized after 3–6 months.

*Total mortality* in HCM (Group 3) was 17/51 (33.3%) of the cases continuing pregnancy with a known follow-up, 13/18 (72.2%) of those with severe ECA, and 2/17 (11.8%) of infants with maternal diabetes.

### 4.3. Statistical Analysis

There were no significant differences between the age at presentation in cases with DCM and myocarditis and relative outcome; cases with fetal hydrops presented poor outcome, but the differences between cases with and without hydrops did not reach statistical significativity. 

In cases with HCM, a negative prognostic factor was apparently the presence of a severe ECA, not reaching statistical significativity with respect to the remaining categories.

## 5. Discussion

Our data confirm a variable spectrum of myocardial disease presenting in the fetal age with different etiology and the possibility of association with extracardiac anomalies both in hypertrophic and dilated forms. Hypertrophic form of cardiomyopathy was more frequent in our series, both in proportion versus the dilated form and with respect to the two previous reports [7, 10], and, particularly, we have found a relevant number of cases with secondary forms. Our figures may be partly due to the referral reasons, operating in the third level centers for prenatal diagnosis where there is a policy to perform the fetal echocardiography also in all cases with extracardiac anomalies. 

The dilated form of cardiomyopathy, either isolated or associated to extracardiac anomalies or infections (myocarditis) was related to a higher perinatal loss than the hypertrophic form, considered on the whole. The outcomes of cases with dilated form of cardiomyopathy was clearly worse in presence of the fetal hydrops, despite the fact that its presence, as well as the earlier age at presentation, did not reach a statistical significativity. An equally high perinatal loss of 82% of cases with DCM was reported by other authors [7]. 

Interestingly, 15.8% of our cases with DCM presented also various extracardiac anomalies and some of these also showed specific features of endocardial fibroelastosis as well as the fetuses with isolated, idiopathic form. 

Family history of DCM was not frequent in our series; it was present in only one of our cases, in a sibling, and of a mild entity. On the other side, the familial DCM may occur more frequently, with a possibility of recurrence in 20–55% [20–22]. Often, there might be underlying metabolic disorders, difficult to precise in the fetal life, as it was in the case of one of our infants; this fact shows how the term of an “idiopathic” form may be incorrect, until a complete evaluation after birth or later in life is done [22].

The problems of a variable timing of presentation of DCM were shown in a fetal-postnatal study on cases with a family history for a nonhypertrophic cardiomyopathy [23], indicating a high recurrence rate of DCM (8/26 = 30.7%), but only half of the cases presented already in utero, in the third trimester (after normal findings at the mid-trimester), while the remaining cases worsened after birth. 

This fact makes it very difficult or impossible to reassure the women referred for the fetal echocardiography for the family history, even in presence of normal fetal exams in the third trimester.

### 5.1. Infections

The infective processes were often put in relationship with the development of dilated cardiomyopathy or endocardial fibroelastosis, following to the finding of antibodies anti-Coxsackie or other viruses in affected patients [7, 9]. Other agents, such as Cytomegalovirus, Rubella virus, parvovirus, and adenovirus, may cause acute myocarditis, with a high perinatal loss. Our cases were all due to Cytomegalovirus and Coxsackie infection and the outcome was poor in those with hydrops, while a few others improved—one already during the fetal life.

*A Recovery of the Left Ventricular Dysfunction.* It, both in cases of idiopathic DCM and after myocarditis, is known to occur, with a variable frequency [24, 25]. Effectively, around one third of our surviving cases with DCM and with myocarditis improved at follow-up, after 3 months–1 year.

### 5.2. Association with Arrhythmias

Tachy- or bradyarrhythmias induce potentially the left ventricular impairment, but there may probably be cases with a major predisposition for the left ventricular impairment in some cases. In our experience (of more than 50 tachyarrhythmias) we have found only one fetus presenting with a specific pattern of DCM. Also the fetuses carrying antibodies anti-Ro, anti-La, passed through placenta from mothers affected with clinical latent immunological disease, may have a predisposition for the development of DCM [26, 27].

*Noncompaction of the Left Ventricle.* It is a particular form of myocardial disease due to a failure of a normal embryological process of myocardial compaction and the cases affected present a variable degree of functional impairment and therefore a variable outcome [14–16]. This condition may be familial, as it occurred in two of our cases—siblings.

*Hypertrophic Form of Cardiomyopathy.* It was more frequent in our series than the dilated form, as mentioned above. HCM is known to be transmitted in some families by autosomal dominant type, with a variable severity. Some of our familial index cases presented a rather mild form of the disease, which increases the difficulty of predicting a possible recurrence later in infancy or in adulthood.

In the fetal life it is difficult to exclude an underlying metabolic disease as glycogenosis or other inborn errors of metabolism found after birth as in one of our cases. Therefore it is imperative to perform a detailed postnatal checkup for metabolic of infective conditions.

We have found during the period of this study also a large number of cases with *secondary forms of HCM,* in presence of some extracardiac anomalies or of the maternal diabetes. Hypertrophy secondary to renal anomalies was described also in another experience [28], and it probably has a similar pathophysiological explanation in the action of angiotensin-renin system, as postnatally. Other extracardiac anomalies, as those of CNS, or multiple anomalies, do not have apparently a clear etiopathogenesis.

Also syndromes as Noonan's can present as HCM, without other associated intracardiac lesions, and in our series we also had a fetus with another rare condition—Thomas's syndrome, defined only after birth. 

A more common form of a secondary HCM is the one related to the maternal pregestational diabetes [29], in which there is an accumulation of glycogen, due to a poorly controlled glucose metabolism. These forms are expected nowadays to be less frequent, with a better control of mothers affected. Our series includes, however, 2 earlier cases who died after birth with features of a marked HCM. Otherwise, these fetuses present usually only minor anomalies of the diastolic flow through the mitral valve due to an impaired compliance [30], but have generally a progressive regression of hypertrophy and normalization after birth, as occurred in all surviving infants of our series.

The evolutivity of HCM is variable, at times worsening, as occurred in two of our cases, one of which needed a heart transplant very early, at 2 months; otherwise some cases can improve or completely regress, mainly the secondary forms, when not related to severe extracardiac anomalies. Echocardiographic follow-up allows nowadays a better assessment of the natural history of affected cases.

## 6. Conclusions

Our data confirm a variable spectrum of myocardial disease in the fetal age, with a possible association of extracardiac anomalies in both forms and more severe prognosis in DCM and myocarditis and in HCM associated with severe extracardiac anomalies. We have to underline the delicacy of the prenatal counseling, in cases with familial history, due to an impossibility to exclude a further presentation of the disease later in infancy or adulthood, as well as a potential association with metabolic or syndromic disorders detectable only after birth. 

As a policy, it is imperative to perform a complete evaluation of the extracardiac organs in utero and a thorough postnatal check-up aiming to exclude underlying conditions. 

The limits of the early detection of myocardial disease are obviously greater in fetuses examined in the second trimester, therefore it is recommendable to repeat the examination in the third trimester and after birth. The cases with family history should also be followed up echocardiographically even later, during infancy and adolescence.

## Figures and Tables

**Figure 3:**
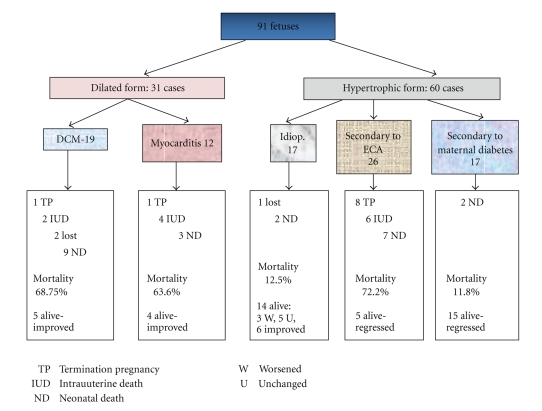
Flow chart summarizing the frequency and the outcomes in the different forms of myocardial disease.

**Table 1:** Data of cases with DCM and myocarditis.

Type of CMP	N.	W.g. at dg.	Assoc. ECAs	Hydrops	Arrh.	Ther. in utero	Outcome in utero-w.g. TP IUD Delivery	Postnatal death	Total death of cases continuing pregn.	Alive/age
DCM LV Idiopathic	Tot. 18 13 (1 Fam., 3 NCM, 5 EFE)	18–35, (median 26)		9	1-SVT 1 ExS	3-dig. 3–4 d	1 TP 2 IUD (24, 30 w.g.) 2 lost, 8 delivered at 33–36 w.g.	4 died		5 alive at 3–10 yrs, improved after 3 months–1 yr
With ECA	5 (3 EFE)		2 renal, 1 CNS, 1 thor.cyst, 1 multiple malform.	3			5 delivered at 34–36 w.g.	4 died		
DCM RV	1	32		1		Dig. 14 d	Delivered by CS at 34 w.g.	Died at day 1		

DCM total	19	18–34	5 ECAs	13 (68.4%)	1 SVT	4 dig.	1 TP, 2 IUD, 2 lost	9 died (4 ECAs)	11/16 (2 lost) =68.75%	5/16 alive (31.25%) and improved

Myocarditis	12	20–37 (median 23)		11 (91.7%) (5 severe, 5 moder., 1 mild)			1 TP, 4 IUD at 23–30 w.g. 7 delivered at 28–40 w.g., median 33	3 died at day 1	7/11 = 63.6%	4 alive, improved

DCM LV, DCM RV: dilated cardiomyopathy of the left ventricle/right ventricle, n.: number, w.g.: weeks' gestation, dg.: diagnosis, Fam.: familiarity, NCM: noncompacted myocardium, ECAs: extracardiac anomalies, malform.: malformations, CNS: central nervous system, thor.: thoracic, arrh.: arrhythmias, SVT: supraventricular tachycardia, ExS: supraventricular extrasystolia, ther.: therapy, dig.: digoxin, d: day, yr: years, TP: termination of pregnancy, IUD: intrauterine death, HF: hydrops fetalis, moder.: moderate, CS: caesarean section, and f-up: follow-up.

**Table 2:** Data of cases with HCM.

Type	N.	W.g. at dg.	Assoc. ECAs/ other conditions	Heart failure	Arrh.	Ther. in utero	Outcome in utero—w.g. TP IUD Delivery	Postnatal death	Total death of cases continuing pregn.	Alive/age
“Idiop.”	17		5 familial history	—	—	—	1 lost 11 delivered at 31-38 w.g.	1–38 d (met.dis)	2/16 with known f-up = 125%	14 alive at 2–14 yrs: *3 worsened*—1 Tx at 2 m; 1 operated at 3 yrs for LVOT-mitral tissue obstruction; 1 needing betablockers; *5 unchanged * (mild-moderate); *6 improved* at 3 m–1 yr.
			12 no familial history					1 prem. born at 28 w.g. died at 15 d.		

Secondary to ECA	26	17–30 Median 22	16 renal, 3 CNS, 7 (skeletal anom., arthrogryposis, Thomas s., Noonan s.)	3 (mild-moder.)			8 TP, 6 IUD at 23–30 w.g., 12 delivered at 28–37 w.g.	7 (1–35 d)	13/18 = 72.2%	5 alive, *regressed *

Secondary to maternal diabetes	17	27–37	12 pregest., 5 gest.	1 (mild)	1 flutter	Dig.*	17 delivered at 37–39 w.g.	2 (2 d, 4 m)	2/17 = 11.8%	15 alive, *normalized* at 3–6 m

HCM total	60	17–39		4 (6.7%)	1	1	8 TP, 6 IUD, 1 lost, 45 delivered	11/51 with known f-up = 21.6%	17/51 with known f-up = 33.3%	34 alive, 2 *worsened*, 6 *unchanged*, 26 *improved*/*normalized *

Idiop.: idiopathic, n.: number, w.g.: weeks' gestation, dg.: diagnosis, ECAs: extracardiac anomalies, anom.: anomalies, CNS: central nervous system, s.: syndrome, arrh.: arrhythmias, SVT: supraventricular tachycardia, ther.: therapy, dig.: digoxin, TP: termination of pregnancy, IUD: intrauterine death, d: day, m: month, yr: year, s.: syndrome, met.dis.: metabolic disease, prem.: premature, gest.: gestational, pregest.: pregestational, moder.: moderate, f-up: follow-up, Tx: heart transplant, and LVOT: left ventricular outflow tract.
